# Analytical characteristics and comparative evaluation of Aptima HCV quant Dx assay with the Abbott RealTi*m*e HCV assay and Roche COBAS AmpliPrep/COBAS TaqMan HCV quantitative test v2.0

**DOI:** 10.1186/s12985-017-0727-3

**Published:** 2017-04-04

**Authors:** A. Worlock, D. Blair, M. Hunsicker, T. Le-Nguyen, C. Motta, C. Nguyen, E. Papachristou, J. Pham, A. Williams, M. Vi, B. Vinluan, A. Hatzakis

**Affiliations:** 1 Hologic Inc., 10210 Genetic Center Drive, San Diego, CA 92121 USA; 2 Department of Hygiene, Epidemiology and Medical Statistics, Medical School, National and Kapodistrian University of Athens, Mikras Asias 75, GR-11527 Athens, Greece; 3 Hellenic Scientific Society for the Study of AIDS and Sexually Transmitted Diseases, Athens, Greece

## Abstract

**Background:**

The Aptima HCV Quant Dx assay (Aptima assay) is a fully automated quantitative assay on the Panther® system. This assay is intended for confirmation of diagnosis and monitoring of HCV RNA in plasma and serum specimens. The purpose of the testing described in this paper was to evaluate the performance of the Aptima assay.

**Methods:**

The analytical sensitivity, analytical specificity, precision, and linearity of the Aptima assay were assessed. The performance of the Aptima assay was compared to two commercially available HCV assays; the Abbott RealTi*m*e HCV assay (Abbott assay, Abbott Labs Illinois, USA) and the Roche COBAS Ampliprep/COBAS Taqman HCV Quantitative Test v2.0 (Roche Assay, Roche Molecular Systems, Pleasanton CA, USA). The 95% Lower Limit of Detection (LoD) of the assay was determined from dilutions of the 2nd HCV WHO International Standard (NIBSC 96/798 genotype 1) and HCV positive clinical specimens in HCV negative human plasma and serum. Probit analysis was performed to generate the 95% predicted detection limits. The Lower Limit of Quantitation (LLoQ) was established for each genotype by diluting clinical specimens and the 2nd HCV WHO International Standard (NIBSC 96/798 genotype 1) in HCV negative human plasma and serum. Specificity was determined using 200 fresh and 536 frozen HCV RNA negative clinical specimens including 370 plasma specimens and 366 serum specimens. Linearity for genotypes 1 to 6 was established by diluting armored RNA or HCV positive clinical specimens in HCV negative serum or plasma from 8.08 log IU/mL to below 1 log IU/mL. Precision was tested using a 10 member panel made by diluting HCV positive clinical specimens or spiking armored RNA into HCV negative plasma and serum. A method comparison was conducted against the Abbott assay using 1058 clinical specimens and against the Roche assay using 608 clinical specimens from HCV infected patients. In addition, agreement between the Roche assay and the Aptima assay using specimens with low HCV concentrations (</= 25 IU/mL by Roche) was tested using 107 clinical specimens.

**Results:**

The 95% LoD was 5.1 IU/mL or lower for serum and 4.8 IU/mL or lower for plasma depending on the HCV genotype. The LLoQ for the assay was 10 IU/mL. Specificity was 100% with 95% confidence intervals of 99.6 to 100% for serum and plasma data combined. The assay demonstrated good linearity across the range for all genotypes. The Precision as estimated by the standard deviation (sd) was 0.17 log or lower across the range of the assay for both serum and plasma. HCV viral load results were compared using the Aptima assay and the Abbott assay giving a slope of 1.06, an intercept of 0.08 and an R^2^ of 0.98. HCV viral load results were compared for the Aptima and Roche assays giving a slope of 1.05, an intercept of −0.12 and an R^2^ of 0.96. Positive and negative agreement for the Aptima assay vs the Roche assay was 89% for low level specimens.

**Conclusion:**

The Aptima assay is a highly sensitive and specific assay. The assay gave comparable HCV viral load results when compared to the Abbott and Roche assays. The performance of the Aptima assay makes it an excellent candidate for the detection and monitoring of HCV.

**Electronic supplementary material:**

The online version of this article (doi:10.1186/s12985-017-0727-3) contains supplementary material, which is available to authorized users.

## Background

Hepatitis C virus (HCV) infection remains an important world-wide health concern with 130 – 150 million people infected [1]. Approximately 75–85% of HCV-infected persons will progress to chronic HCV infection and are at risk for the development cirrhosis, and hepatocellular carcinoma (HCC) [2]. Most people infected with HCV are asymptomatic but the risk of HCC is substantially higher in HCV infected patients compared to HCV negative subjects [3]. The CDC estimates that 1 – 5% of those chronically infected with HCV will die from end stage liver disease due to either cirrhosis or hepatocellular carcinoma [4].

Recently developed direct acting antiviral treatments for HCV can eliminate the virus from almost all patients [5]. The elimination of HCV is measured by a sustained virological response (SVR). SVR is defined as an undetectable HCV RNA plasma/serum concentration at 12 or 24 weeks after treatment completion using a sensitive HCV RNA quantitation assay with a limit of quantitation of under 25 IU/ml [6]. The risks of HCC and mortality are reduced in patients achieving SVR compared to the risks in untreated patients [7].

CDC, AASLD/IDSA and EASL guidelines recommend the use of quantitative HCV RNA testing for pre-treatment and on-treatment patient monitoring and management. Quantitative HCV nucleic acid amplification test (NAAT) results prior to initiating HCV therapy are used to determine the baseline level of viral load, and these results guide decisions for treatment regimens and durations [6, 8, 9]. For diagnosing HCV infections, CDC and AASLD/IDSA recommend initial testing with an HCV antibody test (anti-HCV test). A positive HCV antibody test result is indicative of an active HCV infection or previous HCV infection. An HCV NAAT is recommended to confirm the presence of an active HCV infection or reinfection, after previous viral clearance, in a patient following a positive HCV antibody test result [6, 8]. In addition, HCV NAAT testing is also recommended for patients with negative antibody results, if they are immunocompromised or have been exposed to the Hepatitis C virus within last six months. EASL recommends the use of a sensitive molecular test for HCV RNA for the diagnosis of HCV infection [9].

During HCV therapy, HCV quantitative assays are important tools for determining the effectiveness of HCV treatment. Guidelines from the AASLD and EASL suggest testing HCV RNA not only at baseline, but also periodically during treatment (i.e., 4, 12 weeks) and at completion of treatment (i.e., 8 or 12 weeks) and post-treatment (i.e., 12 or 24 weeks); [6, 9, 10].

This paper presents the data from studies using the Aptima HCV Quant Dx (Aptima; Hologic, Inc., San Diego, CA, USA) assay. This is a quantitative HCV RNA assay for monitoring and diagnosing of HCV infection on the fully automated Panther system. The Aptima assay uses real-time transcription-mediated amplification (TMA) to amplify the RNA target (5’-UTR) of HCV genotypes 1 to 6 [11]. The assay uses a negative and two positive controls to validate accuracy of the results. The assay has a dynamic range of 10 to 100,000,000 IU/mL and uses 500uL of serum or plasma in each reaction. Performance characteristics tested include 95% limit of detection (LoD), lower limit of quantitation (LLoQ), equal genotype detection, linearity precision and specificity. The results of clinical specimens tested with the Aptima assay were compared to those results obtained by the Abbott and Roche assays.

## Methods

### Linearity

The linear range of the Aptima assay was demonstrated by testing panels of HCV armored RNA or clinical specimens diluted in HCV negative human plasma and serum. Panels ranged in concentration from 8.08 log IU/mL to less than 1 log IU/mL. Armored RNA was used to make panels above 6 log IU/mL. A combination of diluted clinical specimens or armored RNA was used to make lower concentrations panels.

### Analytical Sensitivity (Limit of Detection, LoD)

The LoD was determined by testing dilutions of the WHO 2nd International Standard for Hepatitis C Virus RNA (NIBSC 96/798, genotype 1) in HCV negative human plasma and serum. A total of 10 panel members (0.25, 0.50, 1, 2, 3, 6, 8, 10, 12 and 20 IU/mL) were tested over 3 days on 3 Panther systems with 3 reagent lots for a total of 108 replicates per panel member (36 replicates per lot). In addition, The LoD across genotypes was determined by testing dilutions of HCV positive clinical specimens for genotypes 1, 2, 3, 4, 5 and 6 in HCV negative human plasma and serum. The HCV concentration for each clinical specimen was determined by testing with an FDA approved commercially available HCV quantitative assay. Each panel was tested on three Panther systems over 3 days with 3 reagent lots. A minimum total of 60 replicates were tested for each panel member (minimum 20 replicates per reagent lot). Probit analysis was performed on the Aptima results using SAS software to calculate the LoD for 95% detection rates.

### Analytical Sensitivity (Lower Limit of Quantitation, LLoQ)

The LLoQ was determined by testing dilutions of the WHO 2nd International Standard for Hepatitis C Virus RNA (NIBSC 96/798, genotype 1) in HCV negative human plasma and serum. A minimum of 36 replicates of each dilution were tested over 3 days with three reagent lots on 3 Panther systems for a minimum of 108 replicates per dilution. In addition, the LLoQ across genotypes was determined by testing dilutions of HCV positive clinical specimens for genotypes 1, 2, 3, 4, 5 and 6 in HCV negative human plasma and serum. HCV concentration of the clinical specimens was determined using an FDA approved commercially available comparator assay. A minimum of 36 replicates of each dilution were tested over 3 days with each of three reagent lots on 3 Panther systems for a minimum of 108 replicates per panel member. The LLoQ was calculated using a total allowable error of 1 log.

### Analytical specificity

Specificity of the Aptima assay was determined using 100 fresh and 270 frozen HCV negative plasma specimens and 98 fresh and 268 frozen HCV negative serum specimens.

### Precision

To assess precision of the Aptima assay, nine positive panel members made by diluting HCV genotype 1a virus and armored RNA into HCV negative plasma were tested in triplicate by one operator using one reagent lot on one Panther systems over 21 test days.

### Clinical specimen comparisons

Quantitative results for clinical specimens were obtained by running specimens following the manufacturers’ instructions. Quantitative results were obtained for 1058 frozen clinical specimens with both the Aptima and Abbott assays. These specimens represent all six HCV genotypes and cover the full dynamic range of both assays. A subset of these specimens was also tested with the Roche assay. From this subset, 611 gave quantitative results with both the Abbott and Roche assays and 608 gave quantitative results with both the Aptima and Roche assays. Three of the 611 specimens gave an invalid result with the Aptima assay, could not be retested and were excluded from the analysis. Thus, the total number of specimens used for the Aptima vs Roche comparison was 608 and for the Abbott vs Roche comparison was 611. Comparable clinical performance of quantitative measurements was demonstrated by linear regression analysis and Bland Altman plots. The genotype distribution as reported by the vendor or lab providing the specimens is shown in Table 1. One hundred and one specimens did not have a genotype.

**Table 1:** HCV Specimen Genotype & Origin

Country of Origin	Total n	GT 1	GT 2	GT 3	GT 4	GT 5	GT 6	Unknown
Algeria	13	10	2		1			
Australia	33	1	4	22	1		5	
Cambodia	3	3						
Cameroon	25	8	5		12			
Congo	2				2			
Egypt	16	2			13			1
France	89	20	23	30	16			
French Guiana	1			1				
French Polynesia	1				1			
Gabon	9	1			8			
Germany	11	6	1	4				
Greece	406	145	19	179	63			
Guadeloupe	2	1		1				
Italy	85	50	24	3	8			
Ivory Coast	1				1			
Lebanon	1				1			
Libya	1	1						
Luxembourg	2	2						
Madagascar	1	1						
Martinique	1			1				
Mauritius	1	1						
Morocco	37	14	18	3	2			
New Caledonia	1	1						
Reunion Island	1		1					
Russia	12		11	1				
Senegal	1		1					
Spain	34	20	3	8	3			
Thailand	3						3	
Tunisia	29	19	2	1	7			
Unknown	28					17	11	
USA	178	36	11	16	8	2	5	100
Vietnam	30	16	1				13	
Total n	1058	358	126	270	147	19	37	101

### Agreement at low HCV Concentrations

One hundred and seven specimens that gave results at or below 25 IU/mL using the Roche assays were compared. For this analysis, results of “Target Not Detected” for Roche or “Not Detected” for Aptima were interpreted as a negative result. A valid result where HCV was detected or quantified was interpreted as a positive result. Both positive and negative agreement between the Aptima assay and the Roche assay was calculated. Where specimens did not agree and sufficient specimen volume was available, the specimen was tested using the Abbott assay.

The specimens using in the studies were obtained as frozen deidentified remnant samples from the following vendors or labs BioCollection, Miami, Florida, USA;

- Boca Biolistics, Coconut Creek, Florida, USA;
- Cerba Specimen Services, Saint-Ouen l’Aumone, France;
- Discovery Life Sciences, Los Osos, California, USA;
- Hellenic Scientific Society for the Study of AIDS and Transmitted Diseases, Athens, Greece;
- ProMedDx, Norton, Massachusetts, USA;
- SeraCare Milford, Massachusetts, USA;
- SlieaGen, Austin, Texas, USA;
- Tissue Solutions Glasgow, Scotland;
- ViroMed Minnetonka, Minnesota, USA

### Statistical methods

The 95% positivity rates were estimated using Probit analysis using the normal model and analysis software from the SAS Institute, Cary, NC, USA.

The method comparison simple linear regression and Bland Altman analysis were performed using Analyse-It Software, Leeds, United Kingdom.

Linearity was measured using the R^2^ of the Pearson correlation coefficient calculated using Microsoft Excel (WA, USA).

## Results

### Linearity

**Fig. 1:**
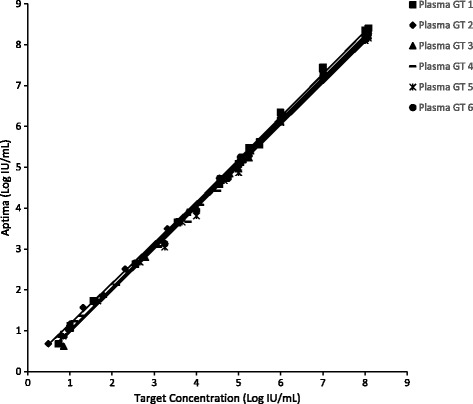
Plasma Linearity for the Aptima Assay. Assay linearity for genotypes 1 to 6 was established by diluting armored RNA or HCV positive clinical specimens in HCV negative plasma from 8.08 log IU/mL to below 1 log IU/mL. Armored RNA was used to make panels above 6 log IU/mL. Clinical specimens were used to make lower concentrations with multiple levels of overlapping concentrations for armored RNA and clinical specimen dilutions. The data for the armored RNA dilutions and the clinical specimen dilutions was combined for each genotype to generate the data shown in the figure

**Fig. 2:**
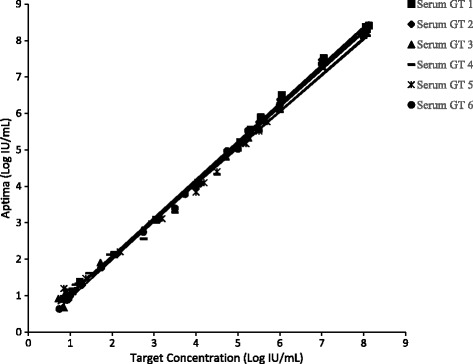
Serum Linearity for the Aptima Assay. Assay linearity for genotypes 1 to 6 was established by diluting armored RNA or HCV positive clinical specimens in HCV negative serum from 8.08 log IU/mL to below 1 log IU/mL. Armored RNA was used to make panels above 6 log IU/mL. Clinical specimens were used to make lower concentrations with multiple levels of overlapping concentrations for armored RNA and clinical specimen dilutions. The data for the armored RNA dilutions and the clinical specimen dilutions was combined for each genotype to generate the data shown in the figure

As shown in Figs. 1 and 2, the Aptima assay displayed a linear response from less than 1 log IU/mL to 8.08 log IU/mL for genotypes 1, 2, 3, 4, 5 and 6 diluted in plasma and serum. The equations of the linear plots for each genotype are shown in Table 2. The R^2^ values for all the linear fits were 1.00. The slopes ranged from 0.99 to 1.06 and the intercepts from −0.12 to 0.17.

**Table 2:** Linear Equations for the Plots shown in Figs. 1 and 2

Matrix	Genotype	Linear Equation
Plasma	1	y = 1.05x - 0.04
	2	y = 0.99x + 0.17
	3	y = 1.02x - 0.02
	4	y = 1.04x - 0.07
	5	y = 1.01x - 0.04
	6	y = 1.03x - 0.02
Serum	1	y = 1.05x + 0.00
	2	y = 1.06x - 0.12
	3	y = 1.02x + 0.05
	4	y = 0.99x + 0.09
	5	y = 1.00x + 0.04
	6	y = 1.05x - 0.11

### Analytical sensitivity of Aptima

Table 3 shows the results of Probit analysis to determine the LoD of HCV genotype 1–6 diluted into either serum or plasma. Three reagent lots were used and the results show the calculated LoD and 95% confidence intervals for each lot separately as well as with all three lots combined. For all genotypes, the calculated LoD was 5 IU/mL or lower. The calculated LLoQ for each HCV genotype is shown in Table 4. The LLoQ varied by genotype from 4 IU/ml for genotype 4 in serum to 10 IU/mL for genotype 6 in serum.

**Table 3:** LoD Probit Analysis Results in Plasma and Serum

HCV Genotype	Plasma - Concentration In IU/mL (95% CI)				Serum - Concentration In IU/mL (95% CI)			
	Lot 1	Lot 2	Lot 3	3 Lots Combined	Lot 1	Lot 2	Lot 3	3 Lots Combined
1 (WHO)	3.3	2.5	4.3	3.4	2.8	3.3	3.9	3.4
	(2.5–5.0)	(1.9–3.9)	(3.3–6.2)	(2.9–4.3)	(2.1 – 4.1)	(2.5 – 5.1)	(3.1 – 5.6)	(2.9 – 4.2)
2	2.8	2.4	2.7	2.7	3.7	3.3	4.0	3.7
	(2.2–3.9)	(1.9–3.4)	(2.3–3.5)	(2.4–3.2)	(3.0–5.3)	(2.6–4.9)	(3.1–5.8)	(3.2–4.5)
3	3.3	4.3	3.1	3.8	2.7	2.9	3.4	3.0
	(2.5–5.2)	(3.2–6.5)	(2.5–4.4)	(3.2–4.8)	(2.2–3.8)	(2.4–4.1)	(2.7–5.1)	(2.6–3.6)
4	2.7	2.4	4.8	3.5	1.3	1.8	2.3	1.9
	(2.2–3.7)	(2.0–3.2)	(3.9–6.3)	(2.7–5.3)	(1.0–2.4)	(1.4–2.5)	(1.7–3.5)	(1.6–2.2)
5	2.0	1.6	2.1	2.0	2.8	2.2	3.2	2.8
	(1.6–3.1)	(1.3–2.4)	(1.7–3.1)	(1.6–2.6)	(2.1–4.9)	(1.8–3.5)	(2.4–5.3)	(2.4–3.6)
6	1.8	3.9	2.6	2.8	2.2	3.8	3.9	3.4
	(1.4–2.9)	(2.8–6.4)	(2.0–3.9)	(2.3–3.6)	(1.7–3.2)	(2.9–5.6)	(3.0–5.7)	(2.9–4.1)

**Table 4:** Summary of LLoQ Results across Genotypes in Plasma and Serum

HCV Genotype	Plasma		Serum	
	LLoQ	LLoQ	LLoQ	LLoQ
	(log IU/ml)	(IU/ml)	(log IU/ml)	(IU/ml)
1 (WHO)	0.82	7	0.93	9
2	0.76	6	0.80	6
3	0.80	6	0.67	5
4	0.82	7	0.65	4
5	0.87	7	0.72	5
6	0.79	6	0.99	10

### Analytical specificity

The results from 370 HCV negative serum and 366 HCV negative plasma specimens are shown in Table 5. Specimens included both fresh and previously frozen specimens. All specimens gave a “Target Not Detected” result for a specificity of 100% (95% confidence intervals 99.2 – 100% for both serum and plasma).

**Table 5:** Specificity for Plasma and Serum Specimens

Aptima HCV Quant Dx	Fresh Plasma	Frozen Plasma	Plasma Total	Fresh Serum	Frozen Serum	Serum Total
(n)	100	270	370	98	268	366
Target Not detected	100	270	370	98	268	366
Specificity (95% CI)	100%	100%	100%	100%	100%	100%
	(97.1–100)	(98.9–100)	(99.2–100)	(97.0–100)	(98.9–100)	(99.2–100)

### Precision

The CVs for inter-day, inter-run and intra-run and total variation were all below 13.3% (Table 6). In all but one case, the largest component of variation was intra-run or random variation. The exception was for the serum panel at 7.16 IU/mL where the intra-run variation was 0.55% but the inter-operator variation and inter-lot variation were 0.70 – 0.73% respectively.

**Table 6:** Precision of the Aptima Assay

Matrix	N^a^	Mean Concentration (log IU/mL)	Inter-Operator		Inter-Instrument		Inter-Lot		Inter-Run		Intra-Run		Total	
			SD	CV (%)	SD	CV (%)	SD	CV (%)	SD	CV (%)	SD	CV (%)	SD	CV (%)
Plasma	98	1.23	0.00	0.00	0.00	0.00	0.04	3.35	0.06	4.54	0.12	9.84	0.14	11.34
Plasma	162	2.06	0.00	0.00	0.05	2.23	0.07	3.20	0.03	1.47	0.10	4.91	0.14	6.88
Plasma	162	3.02	0.01	0.24	0.03	0.89	0.04	1.47	0.01	0.33	0.09	3.08	0.11	3.77
Plasma	162	4.87	0.00	0.00	0.00	0.00	0.03	0.60	0.04	0.86	0.06	1.13	0.10	2.04
Plasma	162	7.16	0.01	0.20	0.02	0.21	0.04	0.52	0.00	0.00	0.05	0.75	0.09	1.27
Serum	132	1.27	0.00	0.00	0.00	0.00	0.07	5.16	0.00	0.00	0.15	12.17	0.17	13.31
Serum	162	2.17	0.04	1.71	0.02	0.99	0.07	3.01	0.05	2.15	0.08	3.53	0.12	5.61
Serum	162	3.09	0.00	0.00	0.02	0.67	0.06	1.79	0.03	0.90	0.07	2.26	0.11	3.44
Serum	161	4.86	0.04	0.78	0.00	0.00	0.08	1.67	0.00	0.00	0.08	1.72	0.13	2.65
Serum	162	7.16	0.05	0.70	0.00	0.00	0.05	0.73	0.02	0.31	0.04	0.55	0.10	1.35

^a^Number of valid results within range of the assay

### Clinical specimen comparisons

**Fig. 3:**
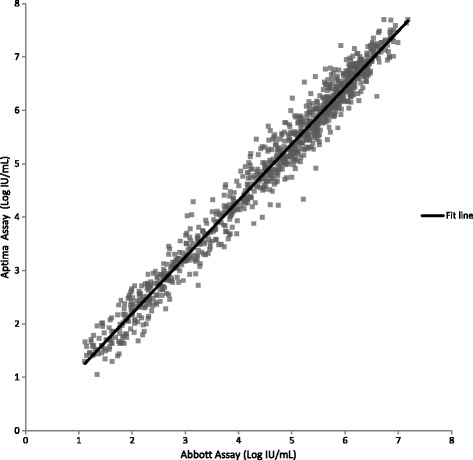
Method Comparison: Aptima vs Abbott. The figure shows results quantifiable in both the Aptima and Abbott assays in log IU/mL for 1058 HCV positive clinical specimens. Linear regression analysis yielded a slope of 1.06 (95% CI: 1.05 to 1.07), intercept of 0.08 (95% CI: 0.03 to 0.13), and R^2^ of 0.98

**Fig. 4:**
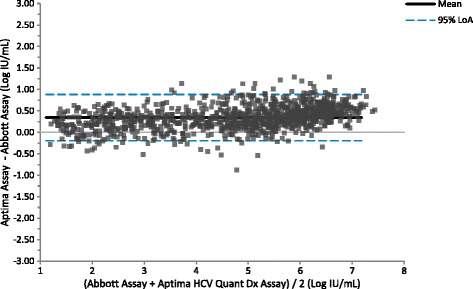
Bland Altman Plot: Aptima vs Abbott. The figure shows results quantifiable in both the Aptima and Abbott assays in log IU/mL for 1058 HCV positive clinical specimens. Bland-Altman analysis was conducted comparing the difference between the Aptima and Abbott assays against the average result for the two assays for each specimen. The average bias was 0.34 log IU/mL with 95% acceptability limits of −0.20 to 0.88 log IU/mL

**Fig. 5:**
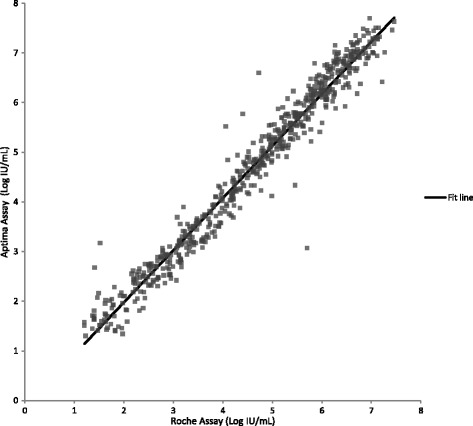
Method Comparison: Aptima vs Roche. The figure shows results quantifiable in both the Aptima and Roche assays in log IU/mL for 608 of the HCV positive clinical specimens. Linear regression analysis yielded a linear regression slope of 1.05 (95% CI: 1.03 to1.07), intercept of 0.12 (95% CI: −0.20 to −0.04), and R^2^ of 0.96

**Fig. 6:**
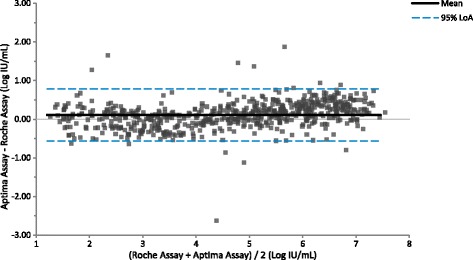
Bland Altman Plot: Aptima vs Roche. The figure shows results quantifiable in both the Aptima and Roche assays in log IU/mL for 608 positive clinical specimens. Bland-Altman analysis was conducted comparing the difference between the Aptima and Roche assays against the average result for the two assays for each specimen. The average bias was 0.11 log IU/mL with 95% acceptability limits of −0.56 to 0.78 log IU/mL

**Fig. 7:**
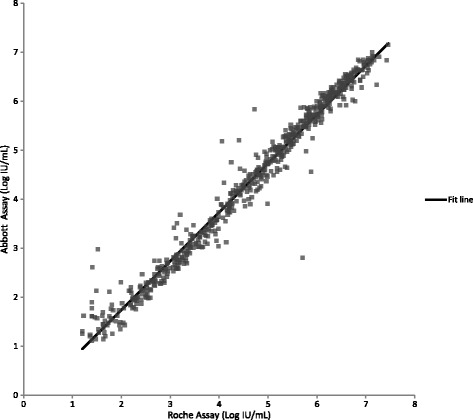
Method Comparison: Abbott vs Roche. The figure shows results quantifiable in both the Abbott and Roche assays in log IU/mL for 611 HCV positive clinical specimens. Linear regression analysis yielded a slope of 0.99 (95% CI: 0.98 to1.01), intercept of −0.25 (95% CI: −0.32 to −0.19), and R^2^ of 0.97

**Fig. 8:**
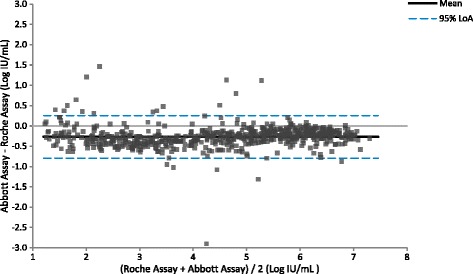
Bland Altman Plot: Abbott vs Roche. The figure shows results quantifiable in both the Abbott and Roche assays in log IU/mL for 611 positive clinical specimens. Bland-Altman analysis was conducted comparing the difference between the Aptima and Roche assays against the average result for the two assays for each specimen. The average bias was −0.27 log IU/mL with 95% acceptability limits of −0.79 to −0.26 log IU/mL

Testing clinical specimens with the Aptima, Abbott and Roche assays allowed a three-way comparison of assay performance to be made. Quantifiable results with both the Aptima and Abbott assays were obtained for 1058 HCV clinical specimens. Linear regression analysis yielded a slope of 1.06 (95% CI: 1.05 to 1.07), intercept of 0.08 (95% CI: 0.03 to 0.13), and R^2^ of 0.98 (Fig. 3, Additional file 1). The slope and intercept were statistically significant (*p* < 0.0001 and *p* = 0.002 respectively). By Bland-Altman analysis (Fig. 4), the average bias was 0.34 log IU/mL with 95% acceptability limits of −0.20 to 0.88 log IU/mL. Quantifiable results with both the Aptima and Roche assays were obtained for 608 HCV positive clinical specimens. Linear regression analysis yielded a slope of 1.05 (95% CI: 1.03 to1.07), intercept of 0.12 (95% CI: −0.20 to −0.04), and R^2^ of 0.96 (Fig. 5, Additional file 1). The slope and intercept were statistically significant (*p* < 0.0001 and *p* = 0.003 respectively). By Bland-Altman analysis (Figure 6), the average bias was 0.11 log IU/mL with 95% acceptability limits of −0.56 to 0.78 log IU/mL. Quantifiable results with both the Roche and Abbott assays were obtained for 611 of the HCV positive clinical specimens. Linear regression analysis yielded a slope of 0.99 (95% CI: 0.98 to1.01), intercept of −0.25 (95% CI: −0.32 to −0.19), and R^2^ of 0.97 (Fig. 7). The slope and intercept were statistically significant (<0.0001). By Bland-Altman analysis (Fig. 8), the average bias was −0.27 log IU/mL with 95% acceptability limits of −0.79 to −0.26 log IU/mL. A summary of the linear regression and bias calculations is shown in Table 7.

**Table 7:** Parameters from Aptima, Abbott and Roche Method Comparisons

Parameter	Aptima vs Abbott	Aptima vs Roche	Abbott vs Roche
n	1058	608	611
R^2^	0.98	0.96	0.97
slope	1.06	1.05	1.00
95% CI for slope	1.04 to 1.07	1.03 to 1.07	0.98 to 1.01
Intercept	0.08	−0.12	−0.25
95% CI for intercept	0.03 to 0.13	−0.2 to −0.04	−0.32 to −0.19
average bias	0.34	0.11	−0.27
95% acceptability limits	−0.20 to 0.88	−0.57 to 0.79	−0.79 to 0.25

### Agreement at low HCV Concentrations

Table 8 shows the agreement between Aptima and Roche results using 107 specimens that gave an HCV results </= 25 IU/mL using the Roche assay. Positive agreement was 89% (47/53) and negative agreement was also 89% (48/54). Six specimens gave a positive result in the Aptima assay and a negative in the Roche assay (Table 9). Sufficient volume was available to test four of the six discordant specimens using the Abbott assay. Two of the four gave detected but not quantifiable results in both Aptima and Abbott assays. The other two gave quantifiable results above 25 IU/mL in both Aptima and Abbott assays. There were six specimens that gave a positive result by Roche but a negative result by Aptima (Table 9). All the Roche results were detectable but not quantifiable. Sufficient volume was available to test two specimens with the Abbott assay and both gave negative results.

**Table 8:** Agreement Between Aptima and Roche for Specimens at or Below 25 IU/mL

		Aptima		
		negative	positive	total
	negative	48	6	54
Roche	positive	6	47	53
	% Agreement	89%	89%

**Table 9:** Discordant Specimens at or Below 25 IU/mL

Aptima Log IU/mL	Abbott Log IU/mL	Roche Log IU/mL
1.97	2.18	TND
2.64	2.59	TND
Detect <10 IU/mL	Detected	TND
Detect <10 IU/mL	Detected	TND
Detect <10 IU/mL	NT	TND
Detect <10 IU/mL	NT	TND
ND	TND	Detected
ND	TND	Detected
ND	NT	Detected
ND	NT	Detected
ND	NT	Detected
ND	NT	Detected

Key

TND, Target Not Detected

ND, Not Detected

NT, Not tested

## Discussion

As recommended by CDC, AASLD/IDSA and EASL guidelines HCV NAAT can be used to confirm the presence of an active HCV infection or reinfection after previous viral clearance in patients with a positive HCV antibody result [6, 8, 9]. Also, current testing guidelines recommend viral load testing with a HCV NAAT prior to initiating HCV therapy to determine the baseline viral load level and to monitor patients while on therapy as well as to detect a sustained virologic response. The data presented in this paper supports the use of the Aptima assay for these purposes.

To be effective as a diagnostic and viral load monitoring test a NAAT assay must quantitate HCV results across a wide dynamic range, be sensitive, specific, accurate, precise and quantify all 6 HCV genotypes similarly. Performance across a large dynamic range is important because HCV RNA levels can reach over 10,000,000 IU/mL [12]. The linearity of the Aptima assay across a wide dynamic range is shown in Figs. 1 and 2. The correlation (R^2^) for samples diluted over a 7 log range in HCV concentration was 1.00. The intercepts for the linear plots were between −0.11 and 0.17 log IU/mL indicating little bias and good linearity.

The sensitivity of the Aptima assay in both serum and plasma is shown in Table 3. Probit analysis to determine the LoD (95% positivity) of HCV genotypes 1–6 diluted into either serum or plasma showed an LoD of 5 IU/mL or lower in all genotypes. In addition, the LLoQ determined in serum and plasma for genotypes 1–6 was 10 IU/mL or lower. This level of sensitivity is important because the end point for therapy of HCV is defined as </=25 IU/mL so an assay must be able to accurately quantitate at levels at or below 25 IU/mL [8]. It is recommended by EASL that for HCV diagnosis, an assay with a lower limit of detection < 15 IU/mL is used [9]. It has also been suggested that testing HCV levels below 50 IU/mL with highly sensitive assays can detect more positive patients and that these patients are more likely to relapse [13]. With a sensitivity of 10 IU/mL (LLoQ), the Aptima assay is able to quantify HCV at the levels needed for effective treatment monitoring and diagnostics.

Specificity is also important to avoid false positive results when diagnosing patients with HCV infections. As shown in Table 5 the specificity of the Aptima HCV was 100% for both serum and plasma specimens.

Sensitivity and specificity of the Aptima assay were further tested using 107 specimens that gave an HCV result </=25 IU/mL using the Roche assay. As shown in Table 8, positive and negative agreement between the Aptima and Roche assays was 89%. Four of the six samples negative by Roche and positive by Aptima, were also tested in the Abbott assay. Two of these were detected but not quantified by Abbott and Aptima. This indicates that these two specimens likely contained very low levels of HCV. However, the other two specimens tested by the Abbott assay, gave results well above the limit of quantitation (2.18 and 2.58 log IU/mL). These specimens gave results of 1.97 and 2.64 log IU/mL respectively with the Aptima assay. This confirms these two results as false negative results by the Roche assay supporting the superior sensitivity of the Aptima assay. There were six results where the Roche assay detected HCV but the Aptima did not. All six specimens gave detectable but not quantifiable results using the Roche assay. Of these six specimens, two had sufficient volume to test using the Abbott assay and both gave “not detectable” results. Data from testing specimens with HCV levels at or below 25 IU/mL suggests that the Aptima assay has better sensitivity than the Roche assay and equivalent or better specificity. The performance of the Aptima assay compared to the reported performance of the Roche and Abbott assays is summarized in Table 10. Based on this data, it is expected that the Aptima assay should show better sensitivity and comparable precision when measuring low levels of HCV.

**Table 10:** Performance Summary of the Aptima, Roche and Abbott Assays

Assay	95% Limit of Detection WHO standard^a^ (IU/mL)	Lower limit of Quantitation (IU/mL)	Linear Range (IU/mL)	Precision (log SD)
Aptima HCV Quant Dx	plasma 4.3 serum 3.9	10	10-100,000,000	0.14 at 2.06 log IU/mL (115 IU/mL)
Abbott RealTi*m*e	plasma 10.5 serum 7.2	12	10-100,000,000	0.10 at 1.96 log IU/mL (91 IU/mL)
Roche Ampliprep/ COBAS Taqman HCV test v2.0	plasma 11 serum 12	15	10-100,000,000	</= 0.09 at 2.48 log IU/mL (300 IU/mL)

^a^Second standard NIBSC 96/798 used for Aptima and Abbott. The third Standard NIBSC 06/100 for Roche Data taken from Aptima (11), Roche [14] and Abbott [15] package inserts

Good precision is an important requirement for an assay used to monitor patients during treatment. A single HCV RNA measurement may be used to confirm that SVR has been reached. The data shown in Table 6 shows that the coefficient of variation for the assay is below 13.3% even down to 1.2 log IU/mL (16 IU/mL). This low variation gives a high degree of confidence that a single RNA measurement using the Aptima assay will provide a reliable result for determining SVR.

The method comparison between the Aptima and Abbott assays used 1058 specimens obtained from over 30 different countries and included genotypes 1–6 representing a highly genetically diverse population of HCV. Statistical analysis of this data set demonstrated a slope of 1.06, an intercept of 0.08 and a correlation of 0.97. The slope was statistically significant and was influenced by slightly higher results for the Aptima assay above 5.5 log IU/mL. Although statistically significant, the magnitude of the difference is not clinically significant. The method comparison between the Aptima and Roche assays used 608 specimens. The slope of the Aptima assay compared to the Roche assay was similar to that seen when comparing the Aptima assay with the Abbott assay (1.05 – 1.06). However, the average bias of the Aptima assay by Bland Altman analysis was lower vs the Roche assay (0.11 log IU/mL) compared to the Abbott assay (0.34 log IU/mL). There were 611 specimens that gave quantifiable results with both the Roche and Abbott assays. A method comparison was conducted with these data and gave a slope of 1.00, an intercept of −0.25 log IU/mL and an average bias of −0.27 log IU/mL. All three assays gave similar HCV viral load results indicating that there is no need to re-baseline patients if switching to the Aptima assay from the Roche and Abbott assays.

## Conclusion

The Aptima assay has excellent sensitivity, specificity and precision. It can measure HCV RNA levels over a large dynamic range. The close agreement to the Abbott and Roche assays suggests that a re-baseline of patients is not required when switching to the Aptima assay for HCV testing. The Aptima assay is an excellent candidate for the diagnosis of HCV and monitoring HCV viral load of patients on HCV therapy.

## Additional file

Additional file 1: Method comparison Aptima Abbott Roche. Supplemental data for VJ publication. (ZIP 16232 kb)

## Abbreviations

AASLD: American Association for the study of Liver Disease
Abbott RT: Abbott RealTi*m*e
Aptima Assay: Aptima HCV Quant Dx assay
CDC: Centers for Disease Control
CV: Coefficient of variation
EASL: European Association for the Study of the Liver
HCC: Hepatocellular carcinoma
HCV: Hepatitis C Virus
IDSA: Infectious Disease Society of America
IVD: In-vitro Diagnostic
LLOQ: Lower limit of Quantitation
LoD: Limit of detection
mL: Millilitre
NAAT: Nucleic acid amplification tests
RNA: Ribonucleic acid
TMA: Transcription Mediate Amplification
WHO: World Health Organization
